# Annotations

**Published:** 1893-09-09

**Authors:** 


### Annotations.

**"My Lord Porridge."**

To a doctor and a dietetician one of the most interesting facts in the daily life of the Scottish people is the profound respect in which they hold oatmeal porridge. It chanced to the present writer, during a recent visit to the "land o' cakes," to have daily set before him at breakfast a plate of porridge of very rare quality. Being an Englishman, and somewhat of an amateur at porridge eating, he had been content with a daily composition of very inferior quality in his own Southern home. But the appetite grows with what it feeds upon, and there seemed to be no reason why as good porridge should not be made in London as in a Scottish village, with a rational cook and good oatmeal. Accordingly, enquiries were instituted as to the proper quarter for securing a regular supply of the very best Scotch oatmeal. The shop of the village was declared to have an excellent pre-eminence for its meal. It was the respect with which the serious spinster in charge of the shop spoke of the cereal which first opened the eyes of the visitor to the place occupied by oatmeal in the Scottish mind. It is clear that this particular grain still constitutes the favourite food of the average Scot. A visit paid to the kitchen to ask the cook for a practical lesson in the art of porridge-boiling deepened the conviction that, next to his Presbyterianism, oatmeal porridge is the chief pride of the Scotchman's life. After fifteen successive mornings of perfect porridge, the Southron was convinced that the Scot is not without justification for his canonisation of oatmeal. It is an appetising, satisfying, and staying food, when made of good meal and perfectly cooked; and it has the advantage of being very economical. That it produces brain, bone, and muscle of the very finest quality Scotland herself is an irrefragable proof. From the point of view of brain, bone, and muscle, we would like to see porridge universally eaten in England. With this object we now disclose the secret of producing one of the finest and most wholesome of all our national dishes. The secret is twofold and very simple. First, get the best of meal; and secondly, let the porridge-maker make the porridge of a judicious consistency, and, above all, stir and boil it thoroughly. We have been assured both by cooks and their mistresses that there are no other "secrets" about porridge-making than these two. In Scotland all classes love the "halesome parritch." We are convinced that if all classes in England would imitate the Scotch in this as they have begun to do in the Royal game of Golf, we should have better appetites, better bones and better brains. A Southron doffs his hat to "my Lord Porridge."

**The Laird of Auchentibber.**

The village of Auchentibber lies in a mining district in Scotland. It is a small place; it numbers only twenty-one cottages, and in these twenty-one houses—more accurately, perhaps, to be entitled huts or hovels—there are, at the date of the present writing, just eighteen cases of typhoid fever—not quite one for each house, but sufficiently near it. The cause of the outbreak is the bad quality of the water, which flows through two ash-pits, and has other opportunities of mingling with miscellaneous sewage before it reaches the inhabitants of Auchentibber. Naturally enough the sanitary authorities have lifted up their voice and said that the water supply of the village must be purified. The expense of this would, of course, fall on the proprietor of the houses, and what does this public-spirited man do? He refuses to interfere with the sewage solution which the villagers drink; but as an alternative he has given them all notice to quit. Twenty-one families, many of them with illness in their midst, are to be turned out because one man refuses to recognise his duties as a citizen and a landlord. Truly we think that a man has a right to do what he likes with his own, but if these cottages are his the lives they hold are not. These lives, already endangered by his carelessness, are to be farther imperilled by his selfish brutality, which simply calculates that after the cottages have been empty for a little new tenants will come who will drink his poisoned water in ignorance of their danger. Indeed, there is no doubt that of the present occupants there are some who will plead to be allowed to remain at any price. They are ignorant people, ready enough to take the fever as a "visitation," and to see no connection between it and the water supply. They are poor people who cannot afford to choose their residence, they must perforce live near their work. Doubtless the landlord knows all this, and reckons on a safe and inexpensive defiance of the authorities. If he succeeds, the more shame to all concerned. For in intent, and in all probability in result, he will be guilty of murder, a wholesale poisoning of the Brinvilliers sort, or something even viler. Is it not possible for him to be indicted on a criminal charge? Such an example, with perhaps a verdict of manslaughter and a good spell of penal servitude would be a useful lesson, and not to the laird of Auchentibber alone. For although his frank indifference to humanity is perhaps unique, there are many proprietors who are quite as unwilling to expend a penny on the well-being of their tenants. They need to be frightened into doing their duty. They might do something if they saw exemplary justice meted out to the laird of Auchentibber.

**How to Detect Quacks.**

We frequently receive inquiries from the public concerning the status and capabilities of men practising as doctors, who have no medical qualification or any other *bonâ fide* fitness for the work they profess to do, and for which they are paid. For example, a teacher in an elementary school wrote to us the other day asking for information about a certain Mr. ——, whose doings were recently exposed in a court of law. The teacher explained that he had had some difficulty in getting together the "rather high fees" claimed by the professed specialist; and he felt, as he expressed it, "that it would be madness" to go on paying these high fees if the so-called "specific" were "worthless." There must be a good many other persons in the same position of unhappy ignorance as this elementary school-master, and they are probably being thoroughly fleeced in the same unscrupulous way. Under these circumstances it may be well for them to know that there are two societies in London, whose business it is to ferret out medical knaves both within and without the ranks of legally authorized practice. These societies do not screen scoundrels merely because they happen to be legally qualified to practise physic. They go for knaves of all sorts because they are knaves. The one society is called The Medical Defence Union, and the other The London and Counties Medical Protection Society. Both the societies give information free of charge to everybody who has a reasonable claim to ask for it. Persons, therefore, like our friend the elementary school-master, who are in any doubt about the *bona fides* of individuals claiming to be specialists and specific-mongers should keep their money in their pockets until they have communicated with one or other of these societies and ascertained the true facts of the case. It is everybody's duty, as it is everybody's interest, to expose pretenders and "frustrate their knavish tricks."

